# G. T. Fechner (1848): Plants as sentient living beings

**DOI:** 10.1080/15592324.2026.2632571

**Published:** 2026-02-18

**Authors:** Giulia Parovel

**Affiliations:** a Department of Social, Political and Cognitive Sciences, University of Siena, Siena, Italy

**Keywords:** Fechner, plant sentience, plant intelligence, plant psychology, animacy from kinematics, Nanna

## Abstract

While Gustav Theodor Fechner (1801-1887) is widely celebrated as the founder of experimental psychophysics, his pioneering work on the psychic life of plants - *Nanna, oder über das Seelenleben der Pflanzen* (1848) - has historically been relegated to the margins as mystical or unscientific. However, a contemporary re-examination reveals that Fechner’s arguments were deeply rooted in empirical observation and inductive reasoning, anticipating current discourse on plant intelligence, learning, and communication. Regarding plant awareness, for instance, Fechner posits that their intimate physical immersion in earth, water, air, and light necessitates that every environmental fluctuation be accessible to their experience. For a sessile organism, survival demands total immersion in the present moment; thus, while the plant may lack the temporal cognitive representations (memory and anticipation) typical of animals, Fechner hypothesizes that its immediate sensorial experience may have reached a degree of intensity even exceeding that of human beings. Overall, Fechner’s perspective offers plant biologists, psychologists, and neuroscientists an original framework to reconceptualize intelligence and perception, suggesting that sentience is an intrinsic property of life itself rather than a mere derivative of neural complexity.

## 1. Introduction

*Whether plants are besouled (beſeelt) or not changes the entire view of nature, and with this question many other things are decided. The whole horizon of the observation of nature expands with the affirmation of this thesis, and even the path that leads to it brings to light points of view that do not enter into the ordinary mode of consideration.* (Transl. from,^1^ Preface)

Gustav Theodor Fechner (1801–1887) is widely recognized as the founder of experimental psychology, having pioneered the fundamental concepts and methodologies of classical psychophysics. In his seminal work,^2^ Fechner demonstrated that the functional relationship between the psychic and the physical could be articulated through precise mathematical laws. This allowed the nascent field of scientific psychology to objectively measure subjective phenomena, such as sensations. Originally trained in medicine, Fechner eventually became a full professor of physics at Leipzig University. Throughout his career, however, he remained deeply influenced by *Naturphilosophie*—a romantic philosophical tradition that viewed nature as a living, integrated whole.^3^

Despite the rapid development of experimental psychophysics within Wundt’s laboratory during that era, Fechner’s primary objective was to propose a new synthesis between the ‘soul-related’ (psychical) and ‘matter-related’ (physical) sciences. His core thesis was the concept of psychophysical parallelism: the idea that consciousness, in some form, is inherent in all of nature. This panpsychic or ‘psychovitalist’ perspective found its most profound expression in his work on the mental life of plants, *Nanna, oder über das Seelenleben der Pflanzen* (1848; Nanna or on the Soul-Life of Plants). In this volume, Fechner investigated whether plants possess a psychic life or a form of consciousness.

Unlike his more famous contributions to psychophysics, *Nanna* (named after the Norse goddess Nanna, whom Fechner identifies as the goddess of flowers) was immediately met with criticism and marginalization. As documented by Hall^4^ and Boring,^5^ the work was dismissed as overly spiritual by psychologists striving to establish a discipline rooted in the objective methods of the natural sciences. Simultaneously, it was rejected as unscientific by botanists of the time (most notably by Schleiden; see.^6^ This historical rejection persists today; Fechner’s pioneering ideas on plants have been largely overlooked and his book is rarely cited in mainstream literature (save for a few recent works such as;^3^^,^^7-10^ furthermore, it is frequently characterized as the byproduct of mystical or religious experiences,^11^^,^^12^ rather than scientific inquiry.

Although *Nanna* is deeply connected with Fechner’s metaphysical project, it deserves to be evaluated on its own terms as a contribution to our *scientific* understanding of plants. Fechner’s investigations into the *Seelenleben* (soul-life, or psychic life) of plants were well-grounded in contemporary empirical research and rational inference.^3^ Fechner utilized scientific methods and inductive reasoning to explore domains of conscious sensitivity and the qualitative aspects of experience.^13^ As he noted in the preface to the first edition:

*What has contributed to increasing the scope of this writing was the desire to combine the exposition of the reasons for our view with an exposition of the factual circumstances which promise to be of relevance for the decision of our question. Unquestionably, the point of view from which this synthesis has been attempted here—should it be deemed valid—can only contribute to increasing the interest which the relevant facts already possess; but even apart from this, this small collection of facts, as material for any sensible reflection on plant life in general, might not be unwelcome to many. In this interest, I have given a somewhat richer material than would have been required for mere sufficiency.* (Transl. from,^1^ Preface)

Notably, despite its significance, to my knowledge, there appears to be no widely accessible or scholarly recognized full English translation of Nanna. Outside of the original German, the work is available in an abbreviated Italian translation^1^^,^^14^ which represents only a fraction of the original 400-page text, and in a complete Spanish translation recently published.^1^^,^^15^

In his era, scientific language was not yet bound by the strictly neutral and impersonal conventions that define modern discourse. Despite its occasionally lyrical and metaphorical prose - reflecting Fechner’s sincere fascination with the mysterious nature of flora - the observations and arguments within the essay anticipate many of the discoveries formulated by the 21st-century ‘plant neurobiology’ movement (e.g.,^16-23^). As Calvo^8^ notes in his Prologue to the Spanish edition of *Nanna*, many of these observations, once purely speculative, are now being validated by modern empirical research. In light of this striking relevance, there is a compelling case for a systematic re-evaluation of his work. This article supports such an effort by outlining the logical frameworks and evidence presented in Fechner’s original writings.

## 2. Signs of consciousness in plants: Fechner’s view

Fechner’s lines of investigation were directed toward identifying the ‘signs’ of psychic life in plants—conceived as a form of consciousness that is necessarily distinct and peculiar relative to human and animal experience. Within his holistic vision of nature, plants and animals are hypothesized as complementary and interdependent systems.

For Fechner, the ‘soul’ (or *psychical life*) is not an entity separate from the physical body; rather, it is defined by the internal fullness and richness of sensations and vital impulses. It serves as a unifying and converging principle for diverse vital experiences, sensations, and drives. It is important to recognize that Fechner did not seek to establish an absolute, dogmatic truth regarding the nature of consciousness. Instead, his objective was to define the boundaries of its plausibility and to dismantle the conceptual obstacles and anthropocentric prejudices that hindered scientific exploration in this domain.

Broadly summarized, at first Fechner's essay deconstructs the most prevalent arguments for the presumed inferiority of the plant kingdom. He identifies these biases in the anthropocentrism of philosophical traditions, the apparent absence of a nervous system, and the misconception of plants as sedentary or still. Subsequently, the author shifts his focus to plant sensitivity. He dedicates several sections to categorizing plant movements—whether induced by instinct, growth processes, or external stimuli. In the remainder of the volume, Fechner formulates conjectures that may be regarded as early ‘thought experiments’, grounded in both analogy and empirical observation. He carefully considers the potential adaptive advantages of the skills developed by plants - necessitated by their condition as sessile organisms - and explores the crucial significance of plant sensitivity in the absence of the higher psychic functions typically attributed to animals.

### 2.1. The anthropocentric prejudice

As articulated by Fechner, the anthropocentric bias is most evident in the traditional view of plants as a subordinate kingdom compared to animals. This perspective imposes a hierarchical scheme upon nature, wherein humans occupy the apex, supposedly possessing all the faculties of ‘lower’ organisms—such as the vital force of plants and the sensitivity of animals—in addition to unique rational faculties. However, Fechner posits that this hierarchy could be replaced by an equally plausible hypothesis: that plants should be understood not as subordinate entities, but as a ‘coordinate’ kingdom, existing alongside the animal sphere with a distinct yet equivalent complexity.

While animals and plants differ significantly in morphology and behavior, they exhibit profound functional analogies in their physiological features. Both are composed of homologous cellular structures, despite differences in higher-level organization; both reproduce via cell division, and both rely on a similar interplay of physiological forces.

Fundamental vital processes - including respiration, nutrient uptake, metabolism, and the internal circulation of substances - are shared across both kingdoms. By highlighting these commonalities, Fechner challenges the assumption that a lack of human-like morphological traits implies a lack of functional or psychic complexity. In this regard, Fechner anticipated modern advancements in phylogeny and biology. Biologists now recognize that plants and animals are sister groups that shared nearly two-thirds of their evolutionary history before diverging into separate lineages.^18^

### 2.2. The absence of a nervous system

The absence of a nervous system continues to be cited—both in popular discourse and within scientific circles—as a definitive reason to relegate the plant kingdom to a status of biological and psychic inferiority. Fechner, however, ingeniously argued that nervous tissue is merely one specific medium for achieving functional ends that might be attained through alternative means. He employed a musical metaphor: while a piano or a violin requires strings to produce a melody, this does not preclude flutes or organ pipes from producing music through entirely different mechanisms. The absence of ‘strings’ (nerves) does not imply the absence of ‘sound’ (sensation).

Furthermore, Fechner posed a provocative teleological question: if plants can perform complex biological imperatives such as nutrition and respiration without a centralized brain or neurons, why should the faculty of feeling be uniquely dependent on them?

Modern research supports this intuition by revealing that plants possess a sophisticated ‘non-neural’ signaling network. They utilize neurotransmitters identical to those found in animal systems - such as glutamate, GABA, and acetylcholine - to mediate long-distance communication via the vascular system.^16^ Furthermore, the propagation of electrical signals, including action potentials, variation potentials, and system potentials, demonstrates that plants have evolved forms of long-distance electrical signaling integrated into their unique modular architecture (for a review, see^24^).

There is no logical necessity to rule out the possibility that plants have evolved specialized structures - distinct from animal nerves - that serve as the substrate for consciousness. Indeed, the organizational blueprint of plants is fundamentally different from that of animals. While animals opted for a centralized system of information processing, argues Fechner, plants evolved a decentralized, modular architecture that may facilitate a different, yet equally sophisticated, form of subjective experience.

### 2.3. The senses of plants

Within a holistic framework - where the diverse manifestations of the natural world are integrated and complementary - the plant kingdom occupies a space distinct from that of animals, yet maintains a relationship of mutual interdependence. From this perspective, plant sensations are intrinsically different from those of humans or animals. As Fechner eloquently states: “Every being is a differently formed sieve, which filters different sensations from nature” (Transl. from,^1^ ch. 4 ‘Teleological reasons’).

Fechner questions the plausibility of a being with such a complex life cycle - one that interacts dynamically with both its external environment and its internal physiology - lacking any form of sensation. He is not suggesting that a lily experiences the world like a sparrow or a cat, but rather that it possesses the subjective experience unique to a lily. This is the sentience of a being that lacks both the centralized processing of a brain and the functional compartmentalization of specialized sense organs.

For instance, when wind strikes a plant, every vibration is transmitted down to the roots. Instead of possessing a localized eardrum, Fechner posits that “the plant is a whole eardrum that the wind hits.” The entire organism acts as a unified sense organ. Regarding floral scents, Fechner suggests they may serve as a form of ‘visiting’ or communication between individuals, essentially acting as the ‘words’ with which flowers encounter one another. This intuitive observation aligns with the modern study of Volatile Organic Compounds (VOCs), which plants use as complex chemical signals to communicate with pollinators, warn neighboring plants of herbivore attacks, and coordinate systemic responses.^22^ What Fechner described as a poetic exchange is now understood as a sophisticated, multi-layered ‘chemical language’ that allows plants to engage in social and environmental interactions.

He further argues that plants must possess a sense of taste to discern between nutrients and observes that light - a resource too vital not to influence sensitivity - is felt across the plant’s entire surface. In Fechner’s view, light is not merely an external stimulus; it becomes internalized: “The splendor of the meadow, adorned with drops of dew, is simply the reflection of the profound inner joy that fills it” (Transl. from,^1^ ch. 4 ‘Teleological reasons’).

Fechner’s reasoning suggests that because of the plant’s intimate, physical immersion in earth, water, air, and light, every fluctuation in these elements must be accessible to its experience. Survival for a sessile organism requires a total immersion in the present moment. While higher cognitive representation requires a temporal dimension (memory and anticipation), the absence of such psychic functions suggests, in Fechner’s hypothesis, that the plant’s immediate sensorial experience may have developed to a degree of intensity far exceeding that of human beings.

### 2.4. Instincts and behavior

According to Fechner, plants manifest the effects of internal impulses through their behavior, albeit in ways that differ fundamentally from animals. These impulses are driven by the biological necessity for light, air, and nourishment. Under the pressure of these drives, plants extend their bodies, directing specific organs toward resources since they are unable to relocate as a whole. Fechner documents, for example, the striking case of a toothwort (*Lathraea squamaria*) which, after falling into the depths of a mine, extended its stem to a length of 20 meters - far exceeding its typical 20-30 cm - in a persistent effort to reach the light.

**Figure 1.**
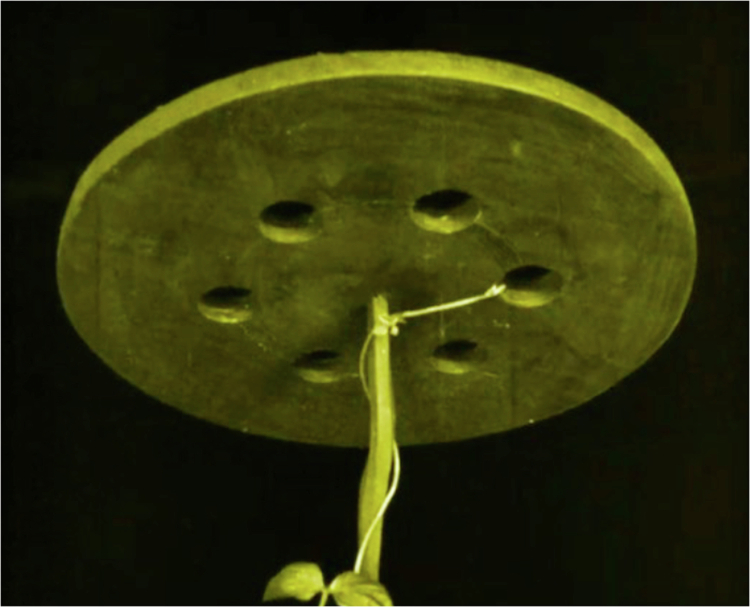
Example of phototropism (from Max Reichmann’s film *The Miracle of Flowers* (*Das Wunder der Blumen, 1926, available at: https://www.youtube.com/watch?v=6jhMPbI3bzc*)

He also describes the sophisticated behavior of a jasmine plant (*Jasminum azoricum*) placed beneath a table perforated with several holes. The stem oriented itself toward the light, navigating toward the nearest aperture. When the table was moved, the stem adjusted its growth trajectory, twining and redirected itself to reach the light through the different holes. Such examples illustrate what modern biology defines as phenotypic plasticity and phototropism (see Figure 1), but which Fechner interpreted as evidence of an underlying psychic drive.

Furthermore, Fechner poses a teleological question: is it possible that plants possess their own intrinsic goals and experience a state of satisfaction when these goals are achieved? He muses whether a water lily might derive a ‘pleasure-like’ sensation from the simultaneous immersion in water and light, or from the act of unfolding its petals by day and retracting them beneath the surface at night. These queries suggest that plants may experience sensations and purposes in a manner entirely their own—mechanisms sufficient to ensure optimal adaptive behavior and promote reproductive success, analogous to the motivational systems observed in the animal kingdom.

### 2.5. The movements of plants

*Nature has this advantage over man: that its works of art—namely, the animals and plants—are self-living beings.* (Transl. from,^1^ ch. 5 ‘The character of plants’)

According to Fechner’s holistic vision, a fundamental correspondence exists between the internal state and the external manifestation of an organism, based on the principle of ‘inner-outer analogy’.^6^ In this view, movement is the primary characteristic of humans and animals, serving as both a means of environmental interaction and an expressive vehicle. The actions of animals—walking, vocalizing, or feeding—directly manifest their psychic and emotional states. In contrast, the psychic character of plants cannot be easily discerned through locomotion, as their movements are typically too slow for human perception. Instead, their agency is expressed through flowering, the release of scents, growth toward light, and the adoption of diverse, adaptive life forms.

**Figure 2.**
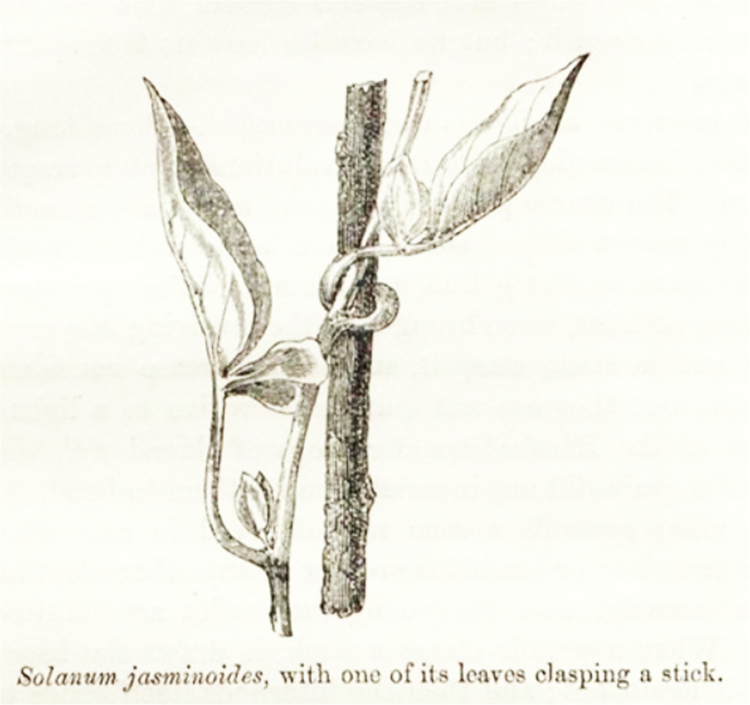
From Darwin’s book *Climbing plants* (1865), *p*. 42. [https://www.darwinproject.ac.uk/letters/darwins-works-letters/climbing-plants].

Plants achieve through growth what animals achieve through movement. Consequently, plants *shape their morphology* in response to multiple environmental factors. Unlike animals, plants must maintain continuous growth and structural adaptation to their surroundings to ensure survival (see Figure 2). Beyond growth, Fechner identifies various other forms of movement, such as the bending, twisting, unfolding, and folding of leaves.

#### 2.5.1. Stimulus-triggered movement and habituation

Fechner describes several ‘movements by stimulation’. For example, if the stamen of a barberry flower (*Berberis vulgaris*) is touched, it executes a rapid movement toward the pistil before slowly returning to its original position. Similarly, the leaves of *Mimosa pudica* collapse upon contact. Crucially, Fechner notes that the more frequently these stimuli occur, the slower the plant reacts. He even observes habituation—a decrease in response to repeated, irrelevant stimuli. This early observation of habituation has been confirmed by modern experimental studies using leaflet closure as the dependent variable, and vertical dropping of the plants as the stimulus,^25^ suggesting a form of non-neural memory and reinforcing the argument that plants do not require a nervous system to perform functions that, in the animal kingdom, are traditionally nerve-dependent.

Fechner cites examples from botanists de Candolle (1778–1841) and Dutrochet (1776–1847) to demonstrate how plants orient themselves optimally relative to light. These cases show that plants employ diverse morphological strategies—such as lateral curvature or the bending of the petiole—to ensure the upper surface of the leaf faces the light source, even when physical obstructions are present.

#### 2.5.2. Circumnutation and the trace of Will

Furthermore, Fechner describes the movements of climbing plants in their search for support - a process later examined in detail by Darwin^26^^,^^27^ as *circumnutation*. He observes how these plants bend their summits while the lower parts remain upright, rotating their horizontally curved portions in a circle to ‘seek out’ an object to grasp. If no support is found, the plant increases the diameter of its search or begins to creep along the ground. He also notes nyctinasty, or ‘sleep movements’, such as the drooping of flowers during the night.

From Darwin’s observations to contemporary research recent data have demonstrated that plant behavior is indeed goal-directed. Many studies examining plant movements have provided evidence that some form of intentionality in plants does indeed exist, providing empirical support for Fechner’s pioneering claims (e.g.,^28–31^). This intentionality, expressed through movements directed toward vital resources, suggests that plants share with humans and animals certain forms of goal-directed behavior allowing for a sophisticated and plastic interaction with their surroundings.^20^^,^^23^^,^^32^

### 2.6. Morphology as the trace of living movement

*Thus, the rose is compared to the blooming girl, and the blooming girl to the rose; the lily stands like a white angel among the flowers, and we gladly compare the pure, angel-like girl back to the lily. So, too, do the vain lady and the tulip, the modest child and the violet, the strong man and the oak, easily and gladly remind us of one another. […] Of course, it would be in vain to want to find all plant characters in human characters or vice versa; flowers and trees are simply not humans. Only here and there does a prevailing connection occur to us, which nevertheless neither fully expresses nor covers the uniqueness of the other.* (Transl. from,^1^ ch. 5 ‘The character of plants’)

In Fechner’s view, the plant psychic life manifests through a continuous interaction between sensation and action. However, this dynamic presents a fundamental perceptual limit for the human observer. Except for rapid-responders like *Mimosa pudica* or carnivorous plants, plant movement is generally too slow for the naked eye.

Anticipating the Gestalt theory on the identity relationship between form and expression^33^; see^34^ for a review), Fechner writes:

*In man and animal, a characteristically different internal structure, a characteristic order and manner of life processes, is attached to the characteristic physiognomy that belongs to them. A different economy of the psychic life requires a different economy of the body as an expression or carrier, and the general line of the form only outwardly indicates to the eye the peculiarly holding-together and closing unity of this inner economy. And exactly as it is with man and animal, so it is with the plant. A human draftsman may indeed execute all his figures, however characteristically different they may be, with hatchings in the same manner; but every different plant form, like every animal form, is internally hatched differently with cells, fibers, tubes; the juices run differently; the forces act differently. And such differences occur not only between different species, like oak, willow, tulip, carnation, but even between different individuals of the same species;* (transl. from,^1^ ch. 5 ‘The character of plants’)

In the 19th century, plant morphology was the only means of tracing the expressive characteristics of plant movement. For Fechner, the conformation of a branch or the direction of a root were not static structures, but the solid trace of a psychic impulse - the result of a will that has become embodied. He regarded plant morphology as a form of **‘**crystallized movement’. This mediated knowledge is evident in Darwin’s studies; unable to witness movement in real-time, Darwin meticulously traced the displacement points of tendrils, root tips, and shoots on glass plates to reconstruct their trajectories.^27^

**Figure 3.**
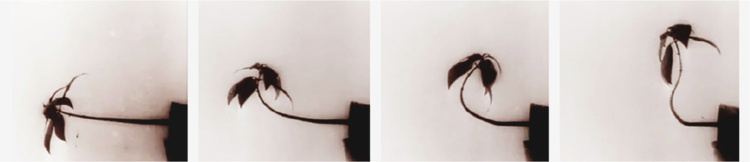
A series of frames from the short film by Wilhelm Pfeffer *Pflanzen Bewegungen* (1898-1900).

A transformative leap in the perception of plants as intentional subjects occurred only with the advent of cinematography and time-lapse photography. While Fechner intuited the active nature of the plant world through careful observation and philosophical reasoning, direct visual evidence of autonomous movement was provided later by botanists such as Pfeffer in 1898 (Figure 3). Time-lapse rendered visible what had previously only been grasped indirectly. Modern technology thus confirmed Fechner’s intuition: the plant is not a passive object, but a sensing subject that actively reacts to its world.

Classic and evocative examples, from both an aesthetic and scientific perspective, include Max Reichmann’s film *The Miracle of Flowers* (*Das Wunder der Blumen, 1926*) and David Attenborough’s acclaimed BBC documentary series, *The Private Life of Plants* (1995). Both cinematic endeavors harness time-lapse cinematography to bridge the temporal gap between human perception and plant life, effectively visualizing the ‘signs of consciousness’ that Fechner could only describe through intuition and careful observation.

**Figure 4.**
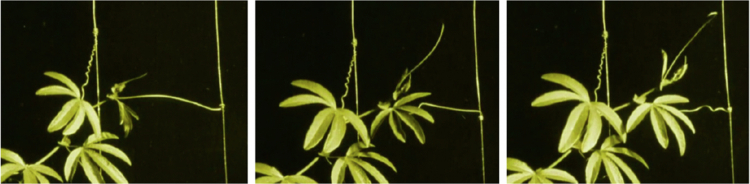
Frames from Max Reichmann’s film. *The Miracle of Flowers* (*Das Wunder der Blumen, 1926, available at:* https://www.youtube.com/watch?v=6jhMPbI3bzc)

In these time-lapse sequences, plants phenomenologically appear as living, intentional creatures that orient their efforts toward interacting with their surroundings (e.g., Figure 4). As current research in visual perception has broadly demonstrated, our visual system is highly efficient at extracting animacy from visual motion and discriminating it from movements caused by passive physical forces (for reviews, see^35-38^). In this view, the time-lapse technique, by compressing time, functions as a methodological bridge that facilitates the detection of potential intentional behaviors, allowing for the observation of plant movements as they interact with environmental cues and neighboring plants.

## 3. Different forms of consciousness: ‘Mental’ versus ‘sensitive’ modes of being

While modern cognitive science distinguishes between non-conscious information processing and phenomenal experience (see^39^^,^^40^) Fechner’s pre-cognitivist approach offers a different nuance. Fechner explicitly acknowledged that plants lack the experience of complex mental or ‘higher’ psychic functions; however, he argued that they have developed an extraordinarily high capacity for sensory-sensitive experience. In his view, plant consciousness is not a ‘diminished’ version of the human mind, but a different form of felt life where the ‘mental’ gives way to an intensified, outward-oriented sensitivity.

*In a more complex organization, higher relations appear above lower ones; and because [in simpler organisms] they are absent, the consciousness related to them is also absent. However, the consciousness of the simplest relations can be just as awake, vigorous, and vivid as that of the higher ones [...] Therefore, even if plants were organized more simply than polyps, there would be no binding reason to consider their soul any less awake than that of those animals; it would only prove a simpler kind of psychic life.* (transl. from,^1^ ch. 12 ‘The Relation of Plants to Animals’)

According to Fechner, in simpler organisms—such as *Hydra viridis* or *Actiniae*—whose bodily structures are relatively uncomplicated, psychic life is directed toward the sensory world and remains devoid of complex mental faculties. However, a lesser degree of structural complexity does not imply a lower intensity or clarity of subjective experience. Fechner argues that living beings cannot be arranged on a simple linear hierarchy; rather, every organic being is superior to any other from a specific vantage point, depending on its unique adaptive ends.

In plants, Fechner posits an even more elementary psychic life, yet one that is as ‘awake’ and precise as that of more developed beings. A crucial requirement for consciousness is the intimate connection between all parts of the organism. While in animals this connection is mediated by a centralized organ—the brain—the parts of plants are equally correlated and concatenated, albeit in a different arrangement. He writes: “If animals are monarchical beehives, then plants are republican anthills”. In line with Fechner’s intuition, recent evidence in the field of animal cognition is challenging the assumed link between centralized brains and complex cognition, indicating that the alternative neural architectures of non-centralized species, such as the moon jellyfish, can support curiosity-like behavior^41^; cf.^40^.

### 3.1. Pure sensibility and the ‘present moment’

According to Fechner, plants may thus be attributed a highly developed ‘sensitive life’, though one limited to the present moment - a state of existence without the temporal dimensions of a past or a future, and thus devoid of ‘higher’ reflective psychic life. This is the soul in its simplest form. Unlike animals, which must look ahead in time to pursue distant goals in space, the plant exists in a germinal condition of pure sensibility. Understood in this way, consciousness should not be considered a separate entity but, as William James would state some decades later, a function of ‘pure experience’, linked to the possibility of ‘knowing’ the surrounding environment. As James noted: “’Consciousness’ is supposed necessary to explain the fact that things not only are, but get reported, are known”.^42^ The perception of the surrounding world does not need ‘ideas’ to arise; it is immediate and primary, anchored in the immediate evidence of sense experience.

The sensibility of the plant constitutes the entire content of its psychic life. The immense volume of external stimuli - combined with the internal multiplicity of the plant’s parts - must arouse a vast array of simultaneous sensations that require integration. As Calvo et al. ^43^ observe, light of different qualities, temperature variations, mechanical inputs of varying strength, water supply variations, gravity, soil complex structures, electrical signals, many volatile and non-volatile chemicals and numerous biological impacts are all perceived, indicating awareness of their environment and often in considerable detail and with considerable sensitivity to even slight changes. Moving beyond an anthropocentric search for sensory equivalents, current scientific inquiry explores the unique and multifaceted perceptual world of plants. Their ability to sense and integrate a broad spectrum of biotic and abiotic signals reveals a complex sensitivity that does not rely on similarity to human frameworks.^8^

The proposal of William James’s radical empiricism has been extensively explored by phenomenology and recently reclaimed by several authors within the philosophy of science to theoretically support the primacy of consciousness (e.g.,^44^). Fechner’s description of this ‘present moment’ consciousness also resembles what Edelman^45^ will define as ‘Primary Consciousness’ regarding the varieties of perceptual awareness that we presumably share with many animals. Primary consciousness, in simple terms, is the capacity to integrate diverse sensory data into a coherent ‘scene’ without the necessity of a narrative self or a historical temporal dimension.

### 3.2. The sensory foundation of consciousness

Thus, returning to Fechner’s conjecture, if the plant’s immersion in pure sensibility places it on a different plane than humans or animals, *it occupies a superior level regarding the development of that sensibility itself*. Unlike the animal, the plant spends its entire life perfecting and increasing its sensory foundation. Within the seed’s readiness to sprout, we find the plant’s singular ‘thought’: a memory of the past and a solicitude for the future of a being similar to itself. Fechner reflects:

*I believe, in fact, that the plant is higher than we are, only in a lower realm. The sensory life (Sinnesleben), even though it is lower, may reach a degree of development which in us is lacking. The life of the senses has only to serve our higher life, whereas it manages its business independently in the plant.* (transl. from,^1^ ch. 4 ‘Teleological reasons’)

For Fechner, the psychic life is not localized at a single point but permeates the entire body. The processes bound to consciousness are ‘psycho-physic movements’- primordial and cosmogenic. Consciousness is the inner unity of a corporeal system, and the preservation of the soul rests upon the solidary cooperation of all bodily activities. This concept of ‘psycho-physic energy’ suggests that consciousness is not a metaphysical essence, but an integral part of the organism’s energy balance - a functional necessity for managing the complex information required for survival and adaptation.

There is only one psychophysical energy; psychic life is not a translation of physical energy into something else, but rather the internal aspect of the same phenomenon. Consequently, the psychic dimension never loses its inherent value.^4^

Fechner’s intuition clearly resonates with the views of many contemporary neurobiologists who, based on research into the cellular foundations of consciousness, suggest that “all life is sentient. Both life and sentience involve self-awareness, evaluation of perceived information and mutually reactive sensory and perceptual functions”^46^; see also^17^^,^^19^^,^^22^^,^^47^).

## 4. Concluding remarks

In recent years, a growing body of scientific evidence has demonstrated that plant behavior is inherently intelligent - characterized as “adaptive, flexible, anticipatory, and goal-oriented”^48^ - rather than a mere product of hardwired instinct (see for reviews^12^^,^^49^). This is substantiated by experiments documenting plant learning, kin recognition, and complex communication. Furthermore, many researchers who support the cognitive capacity of plants suggest the possibility that they are sentient - what Fechner described as *ensouled* (*beseelt*) (see^16^^,^^17^).

The prevailing scientific paradigm posits that consciousness emerges exclusively from complex neural networks; in other words, that the mind is a functional derivative of the brain (cf.^40^, for a multifaceted discussion on the current plant cognition debate). Interestingly, a re-examination of Fechner suggests a compelling and underexplored alternative: the hypothesis that cognition and consciousness may be regarded as two eventually overlapping yet biologically distinct systems. Plants may not require higher-order cognitive processes to *feel* and *act*; instead, their heightened and amplified sensitivity (*sentience*) may be sufficient for autonomous agency and intentionality. Unlike animals, plants are not organized to navigate their environment through locomotion, complex memory, or symbolic prediction, but rather *to sense* minute fluctuations across environmental dimensions and respond with metabolic and morphological precision.

In Fechner’s perspective, consciousness, understood as *sentience*, should be viewed as a faculty intrinsically linked to life itself. Rather than a late by product of evolution or a consequence of symbolic-cognitive complexity, it is the foundational element that enables and sustains life, even in the most elementary organisms (cfr.^17^^,^^18^^,^^50^). The alternative, as Fechner warned, is a world as arid as a desert, containing only isolated fragments of subjective life. “Is it not more beautiful and fulfilling to think of the trees in the forest as living beings shining toward the sky, rather than merely diffusing light as they burn?”,^1^ ch. 4 ‘Teleological reasons’). Such a view is not only counterintuitive but also scientifically improbable.

As Calvo et al. ^43^ argue, consciousness clearly influences behavior and serves a vital biological function: biological awareness is simply a readiness to respond to particular patterns of stimulation both from inside the organism and from outside. Plants are demonstrably aware of the external world, responding meaningfully through adaptive behavioral changes.^16^^,^^51^

Rather than being a mere product of mysticism or personal crisis,^11^ Fechner’s pioneering proposal had the sole limitation of being too far ahead of its time. It resonates with contemporary efforts to advance non-brain-centric and non-representational accounts of consciousness and certainly deserves to be rediscovered and revisited by plant biologists, psychologists, and neuroscientists alike (see^52^).

His perspective finds common ground with theoretical frameworks such as the 4E cognition program^49^ and ecological approaches to plant behavior.^53-55^ These viewpoints emphasize the value of observing plant life without theoretical bias or anthropocentric constraints—moving beyond the expectation that plant behavior must mirror human patterns to be deemed ‘conscious’.^56^

Given the technological potential of current high-resolution imaging and recording tools, a phenomenological and unprejudiced approach may pave the way for discoveries that are currently inconceivable. For scientific psychology, to include plant behavior within the field of comparative psychology represents an original and stimulating frontier.^49^^,^^57^ It offers the opportunity to challenge and reconceptualize fundamental constructs—such as intelligence, consciousness, and perception—thereby clearing the path for new theoretical formulations in the study of life and mind^58^^,^^59,^^60^, providing a significant contribution to the wider area of current research on the world of these fascinating and mysterious living beings.
